# Topical Bimiralisib Shows Meaningful Cutaneous Drug Levels in Healthy Volunteers and Mycosis Fungoides Patients but No Clinical Activity in a First-in-Human, Randomized Controlled Trial

**DOI:** 10.3390/cancers14061510

**Published:** 2022-03-15

**Authors:** Selinde S. Wind, Manon A. A. Jansen, Melanie Rijsbergen, Michiel J. van Esdonk, Dimitrios Ziagkos, Wing C. Cheng, Tessa Niemeyer-van der Kolk, John Korsten, Agnieszka Gruszka, Debora Schmitz-Rohmer, David Bonnel, Raphael Legouffe, Florian Barré, Marcel W. Bekkenk, Ellen R. M. de Haas, Koen D. Quint, Melanie Rolli, Henk Johan Streefkerk, Jacobus Burggraaf, Maarten H. Vermeer, Robert Rissmann

**Affiliations:** 1 Centre for Human Drug Research, 2333 CL Leiden, The Netherlands; swind@chdr.nl (S.S.W.); mjansen@chdr.nl (M.A.A.J.); m.rijsbergen@hotmail.com (M.R.); mvesdonk@chdr.nl (M.J.v.E.); ziagkosd@gmail.com (D.Z.); wingchicheng@gmail.com (W.C.C.); tvdkolk@chdr.nl (T.N.-v.d.K.); kb@chdr.nl (J.B.); 2 Department of Dermatology, Leiden University Medical Center, 2333 ZA Leiden, The Netherlands; k.d.quint@lumc.nl (K.D.Q.); m.h.vermeer@lumc.nl (M.H.V.); 3 Charles River Laboratories Den Bosch B.V., 5231 DD Den Bosch, The Netherlands; john.korsten@crl.com (J.K.); agnieszka.gruszka@crl.com (A.G.); 4 PIQUR Therapeutics AG, 4057 Basel, Switzerland; debora.schmitz.rohmer@gmail.com (D.S.-R.); melanie.rolli@gmail.com (M.R.); hendrik.streefkerk@gmail.com (H.J.S.); 5 MS Imaging Department, ImaBiotech, 59120 Lille, France; bonnel.david@imabiotech.com (D.B.); legouffe.raphael@imabiotech.com (R.L.); barre.florian@imabiotech.com (F.B.); 6 Amsterdam University Medical Centers, 1105 AZ Amsterdam, The Netherlands; m.w.bekkenk@amsterdamumc.nl; 7 Erasmus Medical Center, 3015 GD Rotterdam, The Netherlands; e.r.m.dehaas@erasmusmc.nl; 8 Leiden Academic Centre for Drug Research, Leiden University, 2333 CC Leiden, The Netherlands

**Keywords:** CTCL, mycosis fungoides, pharmacokinetics, bimiralisib, PQR309, PI3K/mTOR

## Abstract

**Simple Summary:**

Primary cutaneous T-cell lymphomas (CTCLs) are a group of lymphomas that present in the skin without extracutaneous localizations at diagnosis. Recent studies in clinical and translational research augmented our understanding of the pathogenesis and pathophysiology of different subtypes of CTCL, enabling the identification of novel therapeutic drug targets. In this study, the primary focus is on bimiralisib gel 2%, a dual pan-class PI3K/mTOR inhibitor, and its potential to inhibit the PI3K/AKT/mTOR signaling pathway as a novel therapeutic target in CTCL.

**Abstract:**

Mycosis fungoides (MF) is a subtype of CTCL with a low incidence and high medical need for novel treatments. The objective of this randomized, placebo-controlled, double-blinded, first-in-human study was to evaluate safety, efficacy, cutaneous and systemic pharmacokinetics (PK) of topical bimiralisib in healthy volunteers (HVs) and MF patients. In this trial, a total of 6 HVs and 19 early-stage MF patients were treated with 2.0% bimiralisib gel and/or placebo. Drug efficacy was assessed by the Composite Assessment of Index Lesion Severity (CAILS) score, supported by objective measuring methods to quantify lesion severity. PK blood samples were collected frequently and cutaneous PK was investigated in skin punch biopsies on the last day of treatment. Local distribution of bimiralisib in HVs showed a mean exposure of 2.54 µg/g in the epidermis. A systemic concentration was observed after application of a target dose of 2 mg/cm^2^ on 400 cm^2^, with a mean C_avg_ of 0.96 ng/mL. Systemic exposure of bimiralisib was reached in all treated MF patients, and normalized plasma concentrations showed a 144% increased exposure compared to HVs, with an observed mean C_avg_ of 4.49 ng/mL and a mean cutaneous concentration of 5.3 µg/g. No difference in CAILS or objective lesion severity quantification upon 42 days of once-daily treatment was observed in the MF patient group. In general, the treatment was well tolerated in terms of local reactions as well as systemic adverse events. In conclusion, we showed that topical bimiralisib treatment leads to (i) meaningful cutaneous drug levels and (ii) well-tolerated systemic drug exposure in MF patients and (iii) a lack of clinical efficacy, in need of further exploration due to numerous unknown factors, before depreciation of topical bimiralisib as a novel therapeutic drug for CTCLs.

## 1. Introduction

Primary cutaneous T-cell lymphomas (CTCLs) are a heterogeneous group of T-cell lymphomas characterized by extravasation and migration of malignant T lymphocytes to the epidermis and the papillary dermis. CTCL is, with an incidence of 10.2 per million people [1], an ultra-orphan disease. Mycosis fungoides (MF) is the most common type of CTCL and comprises almost 50% of all primary CTCL cases [2]. Early-stage (stage IA-IIA) MF manifests as erythematosquamous patches and/or plaques, typically on sun-protected areas affecting <10% BSA (stage IA) or ≥10% BSA (stage IB), with possible reactive lymph nodes (stage IIA). It usually exerts a slowly progressive course with progression from plaques to tumors (≥stage IIB) and extracutaneous involvement (≥stage IVA) in a minority of patients. Pruritus and esthetic complaints are experienced as most bothersome by the patients and can lead to a significantly reduced quality of life [3]. The most frequently used therapies for early-stage MF include phototherapy (PUVA/UVB), chlormethine gel and/or topical corticosteroids. Whilst these therapies are very effective in suppressing early-stage MF, chronic use can be limited by side effects such as skin atrophy, phototoxicity, dermatitis, itching and photoaging with an increased risk for skin malignancies (e.g., squamous cell carcinoma with PUVA phototherapy [4,5]) over time. Given the chronic character of MF that requires long-term treatment, there is a high medical need for efficacious topical therapies with minimal side effects.

Bimiralisib is a potential novel topical treatment option for patients with MF. It is a small molecule acting as dual-action phosphoinositide 3-kinase (PI3K) and mammalian target of rapamycin (mTOR) inhibitor, best described as a selective pan-class I PI3K-inhibitor with balanced activity against mTOR, which is known to play a significant role in the pathogenesis of MF. The PI3K/v-akt murine lymphoma viral oncogene homolog (AKT)/mTOR signaling cascade serves many (patho)physiological functions and is one of the major cancer signaling pathways playing a critical role in regulating cancer cell growth, survival and proliferation [6,7,8,9,10,11,12]. Witzig et al. demonstrated that CTCL cell lines have activated mTOR signaling compared to normal T cells and that the mTOR complex 1 inhibitor everolimus has antitumor activity in vitro [13]. Activation of the PI3K/AKT/mTOR pathway in MF is associated with tumorigenesis in MF patients. Evaluation of clinical features and in situ PI3K and phosphatase and tensin homolog alterations on the (epi)genetic and protein level found that increased expression of phosphorylated AKT was correlated with poor prognosis in patients with plaque stage MF, and poor survival for an entire MF patient cohort [14]. In addition, *Horwitz* et al. demonstrated promising clinical activity with 3 complete responders and 6 partial responders by modified Severity-Weighed Assessment Tool (mSWAT) and an acceptable safety profile for the oral PI3K inhibitor duvelisib in a study with 19 CTCL patients [15]. However, no topical PI3K inhibitor has been evaluated so far in CTCL patients. Multiple clinical studies, including studies on 233 patients with various malignancies (including lymphomas), demonstrated potential clinical activity of oral or intravenous bimiralisib. However, all studies also observed substantial systemic adverse events (AEs, e.g., rash, anemia, neutropenia, depression, increased ALT and AST, hyperglycemia) [16,17,18,19,20,21]. To elude systemic AEs, as well as address the medical need for new topical therapies for MF, a gel formulation of bimiralisib was developed. The objective of this study was to explore the safety, efficacy and pharmacokinetics (PK) of topical bimiralisib in healthy volunteers and patients with early-stage MF. Therefore, we conducted a randomized, placebo-controlled first-in-human trial in both volunteer groups with a strong emphasis on systemic and cutaneous PK.

## 2. Materials and Methods

### 2.1. Study Design, Randomization and Treatments

The study consisted of two parts. Part A was an open-label, single-dose study in 6 healthy volunteers (HVs) to evaluate the safety, tolerability and pharmacokinetics (PK) of topical bimiralisib. Part B had a randomized, double-blind, placebo-controlled, single-center, proof-of-concept design to characterize safety and tolerability, clinical efficacy and PK of topical bimiralisib in 19 patients with early-stage CTCL-MF (stage IA-IIA). The study was carried out from June 2019 until August 2019 (Part A) and October 2019 to April 2020 (Part B) at the Centre for Human Drug Research, Leiden, The Netherlands, with the Declaration of Helsinki as the guiding principle for trial execution. The study was approved by the independent Medical Ethics committee “Medisch Ethische Toetsingscommissie van de Stichting Beoordeling Ethiek Biomedisch Onderzoek” (Assen, The Netherlands) before trial execution and registered in the EudraCT database with identification number 2019-001383-30. All participants gave written informed consent before any study-related activities.

The healthy volunteers in part A were administered 2 mg/cm^2^ topical bimiralisib 2% on 400 cm^2^ and 2 mg/cm^2^ vehicle gel (serving as placebo with identical appearance) on 100 cm^2^ skin of the back once daily by the study physician for 21 consecutive days, all administered at the study site. The 19 patients in part B were randomized 1:1, with 9 assigned to active and 10 to placebo. Randomization was predefined and performed in SAS version 9.4 (SAS Institute, Cary, NC, USA) by an independent statistician, using the SAS code for the parallel, two-treatment randomization with block size 2. The investigators, study personnel, sponsor and patients were blinded for allocated treatment throughout the study. Patients randomized in the treatment group applied topical bimiralisib 2%, once daily for 42 consecutive days, whereas the control group applied the vehicle gel. During the treatment period, the patients applied the bimiralisib or vehicle themselves at home after training and instructions on day 1. After 42 days, patients with ≥15% lesion improvement by CAILS could enter a blinded extension period in which treatment was continued until day 84.

At baseline of part B, one to three MF lesions (target lesions) were chosen to define a 150–200 cm^2^ treatment area. Other MF lesions were left untreated in this study. Emollients, i.e., unguentum leniens, were distributed to patients and allowed for daily use on the skin, barring the target lesion(s). In part B, patient visits to the study site were scheduled for days −42 (run-in period), 1, 14, 28, 42 (end of treatment for all patients with <15% lesion improvement), 56 and 70 (end of study). Patients continuing with the extended treatment period had three extra visits on days 84 (end of treatment), 98 and 112 (end of study). Study details are provided in the assessment schedule (Table 1, Part B).

### 2.2. Participants

Healthy volunteers were included in part A if they were males of ≥18 years old with Fitzpatrick skin type I–IV. Significant skin disease, history of hypertrophic scarring and a period shorter than 2-week washout for all topical treatments in the treatment area were exclusion criteria.

Male and female MF patients were included in part B if they were ≥18 years and had no clinically significant or unstable disease other than MF. A confirmed histopathological diagnosis of MF stage IA or IB within the last 5 years and having at least 1, 2 or 3 target lesions with a total combined size of 150–200 cm^2^ were required. Topical and systemic treatments for MF had to be stopped prior to the first application of the study drug within two and four weeks, respectively.

All participants of reproductive age (part A and B) were obliged to use double effective contraception during study execution and at least 90 days onwards. Patients with a known hypersensitivity to any of the excipients of bimiralisib gel and patients that required systemic therapy for MF or other active malignancies were excluded. Furthermore, for safety reasons, pregnant or lactating women and patients with a known hepatitis or HIV infection were not allowed to participate in the trial. For an overview of all inclusion and exclusion criteria for HVs and MF patients, see Table S1.

### 2.3. Clinical Efficacy and Pharmacodynamics

The primary endpoint was to evaluate the efficacy of bimiralisib versus placebo after 6 weeks of treatment by the change from baseline on Composite Assessment of Index Lesion Severity (CAILS). Clinical efficacy was assessed by the CAILS per target lesion, which is a score derived from individual scores for erythema, desquamation, plaque elevation, hypo- or hyperpigmentation and lesion size. Change from baseline of the combined CAILS score for all target lesions was used to calculate the objective response rate (ORR), i.e., the number of patients with complete response (CR: 100% clearance of target lesions from baseline) and partial response (PR: 50%–99% clearance) divided by the total number of patients in the respective treatment arm. Stable disease was classified as <25% increase to <50% clearance in target lesions from baseline, whereas progressive disease implied ≥25% increase in target lesions by CAILS [22]. Target lesions were extensively characterized by the assessment of skin perfusion with laser speckle contrast imaging (LSCI, PeriCam PSI NR system, Perimed, Sweden); skin morphology (blood flow) by optical coherence tomography (OCT, VivoSight, Michelson Diagnostics, UK); and erythema, roughness and edema by a multispectral camera (Antera 3D, Miravez, Ireland). The Scarlet Red app (ScarletRed Vision, Vienna, Austria) [23] was used by patients at home for daily photo documentation and erythema quantification of a single target lesion.

### 2.4. Safety and Tolerability

The secondary endpoint was to characterize local tolerability, safety and systemic exposure of bimiralisib by application site assessment using the Local Irritation Grading Scale (LIGS), adverse events (AEs), safety measurements and systemic pharmacokinetics.

The LIGS, a composite score of erythema, edema and desquamation, was used to assess the application site for safety or tolerability issues. In addition, safety and tolerability were evaluated by monitoring of AEs, physical examinations, vital signs, 12-lead ECGs and laboratory tests.

### 2.5. Treatment Compliance and Exposure

A validated mobile-phone eDiary application was used to monitor patient compliance for daily administration of the study drug. The application comprises a notification and photo function enabling documentation of date and time with each gel application [24]. Individual tubes used for home application were weighed every study visit to determine the average applied dose per day per patient.

### 2.6. Pharmacokinetics

Blood samples for determination of bimiralisib levels in plasma of HVs were collected predose and at 1, 3, 6 and 24 h on days 7, 14 and 21 of application in part A. In part B, PK blood samples from patients were collected predose and at 1, 2, 3 and 6 h after dosing on day 42 (or on day 84 in case of participation in the extension study) of topical application.

Pharmacokinetics in plasma were analyzed with a validated ultra-performance liquid chromatography–tandem mass spectrometer method (UPLC-MS/MS) with a lower limit of quantification of 1.00 ng/mL of plasma.

**Figure 1:**
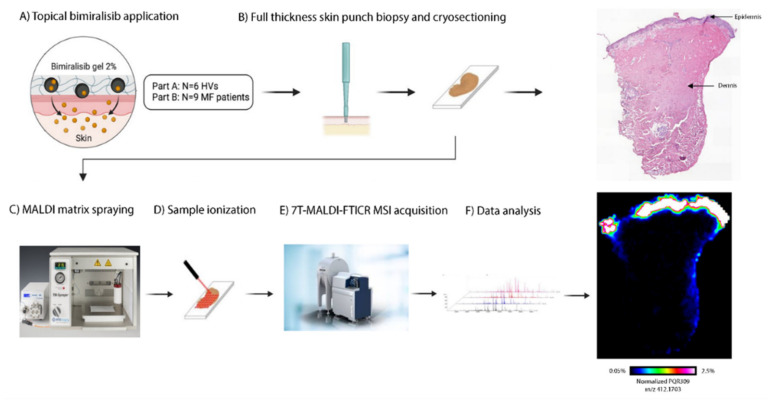
Method of obtaining cutaneous pharmacokinetics of bimiralisib in HVs and early-stage MF patients. (**A**) Bimiralisib gel 2% is applied topically, in part A at the back of six HVs and in part B at 1–3 target MF lesions. (**B**) A full-thickness skin punch biopsy was taken on day 21 and day 42/84, respectively, for HVs and MF patients. Skin punch biopsy was snap-frozen within 5 min, processed and cryosectioned. A single section was used for H&E staining. (**C**) MALDI matrix spraying. (**D**) Prepared samples were ionized by a laser beam. (**E**) Seven tesla Matrix-assisted laser desorption/ionization Fourier transform ion cyclotron resonance mass spectrometry imaging (7T-MALDI-FTICR MSI) acquisition. (**F**) Acquired data are analyzed and MALDI images are acquired.

To investigate cutaneous pharmacokinetics in both HVs and patients, 3 mm skin punch biopsies of the treatment area were taken 6 h after the last topical application (day 21 in part A and day 42/84 in part B, respectively) and snap-frozen within 5 min after collection. Skin punch biopsies were shipped to ImaBiotech (MS Imaging Department, Lille, France) and analyzed using matrix-assisted laser desorption/ionization mass spectrometry imaging (MALDI-MSI) to spatially visualize pharmacokinetics of bimiralisib throughout the biopsy (Figure 1).

Pharmacokinetics in tissue were analyzed with a validated 7T-MALDI-FTICR method with a lower limit of quantification of 62 ng/g of tissue and an assay sensitivity of 95 ng/g of tissue (lower limit of detection). Pharmacokinetic data below the lower limit of quantification but above the limit of detection were interpolated and used in the analysis.

### 2.7. Statistics

A sample size of nine patients per treatment group was estimated based on analysis of data available in relevant literature regarding PI3K inhibitors in early-stage CTCL patients [25]. To provide a power of 80% to demonstrate the superiority of bimiralisib over placebo with a between-treatment difference of 42%, a common SD of 30% was chosen, using a two-group t-test with a two-sided alpha of 0.05 (Table S2).

All safety and statistical calculations were conducted with SAS version 9.4 for Windows (SAS Institute, Cary, NC, USA). PK parameter calculations were conducted with R version 4.0.3 for Windows (R Foundation for Statistical Computing, Vienna, Austria). All efficacy endpoints were analyzed for both the per protocol set and full analysis set, using a mixed model analysis of covariance (ANCOVA) with treatment, time and interaction of treatment by time as fixed factors; baseline as covariate; and subject as random factor.

Pharmacokinetic data were visualized for each subject and part separately. Due to differences in surface areas and applied doses between part A and part B, pharmacokinetic data were normalized to allow for the exploration of differences in exposure between HVs and patients after log transformation. For exploratory purposes, the applied dose was stratified by part and by number of lesions. The average concentration (C_avg_) per subject was calculated based on all pharmacokinetic data of an individual combined.

## 3. Results

### 3.1. Patient Characteristics

A total of 17 HVs were screened in part A, of whom 6 were enrolled in the trial (Figure S1). All HVs received both treatments for 21 consecutive days (Table 1, Part A) in an open-label fashion. Baseline characteristics were comparable between individuals, and all HVs completed the study (Table 2).

**Figure 2:**
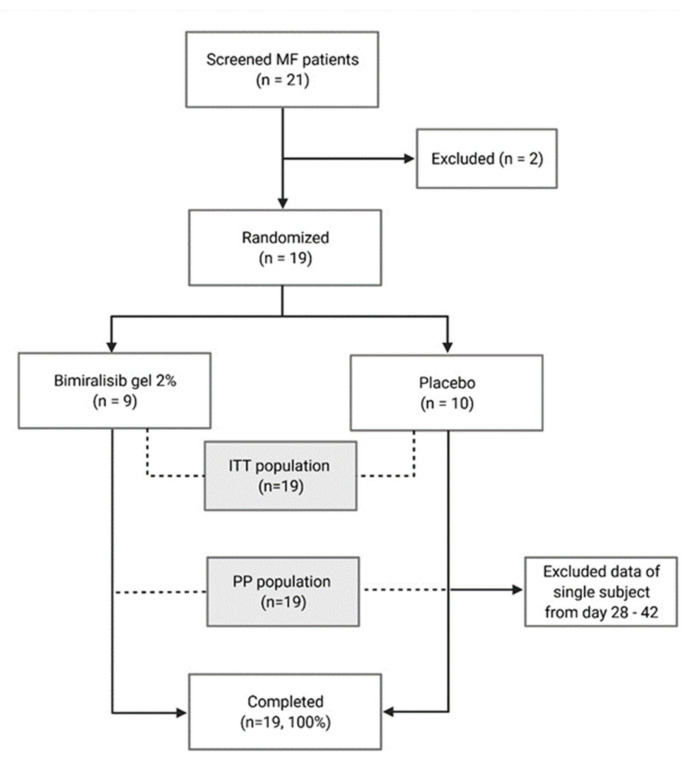
Study flow chart of part B with MF patients. ITT = intention to treat; PP = per protocol. Two patients were excluded for not meeting the inclusion criteria. Data of a single patient between days 28 and 42 were excluded due to a pharmacy dispensing error.

In part B, 21 MF patients were screened, of whom 19 patients were randomized (Figure 2). Ten patients were treated with topical placebo, and nine subjects with topical bimiralisib, on a skin surface ranging from 150 to 200 cm^2^ for 42 days.

All enrolled patients completed the study; however, data from day 28 to day 42 of a single subject were excluded and the subject was replaced after day 28. Baseline and disease characteristics were comparable (Table 2). Two subjects in the placebo group and three subjects in the bimiralisib group continued treatment after 42 days in the extension period of the study. Treatment compliance was on average 98.1% (range 92.7%–100%) in the bimiralisib group and 96.4% (range 88.1%–100%) in the placebo group.

### 3.2. Safety and Tolerability

In part A, all six subjects (100%) experienced one or more adverse events (AEs, Table S3). The most frequent AE, reported by five subjects (83.3%), was dryness at the application site, which was reported for both placebo-treated and bimiralisib-treated skin areas. Other AEs at the application site included acne (n = 3, 50%) at the bimiralisib area and discomfort, pruritus and pustules each occurring at the placebo area in the same subject (n = 1, 16.7%).

In part B, nine patients (90%) in the placebo group and six patients (66.7%) in the bimiralisib group experienced at least one AE (Table S4). The most frequent AEs were headache (n = 6) and pruritus (n = 3), of which seven events (7/9, 77.8%) occurred in the placebo group. The most frequently reported AE for patients in the bimiralisib group was myalgia (2/9, 22.2%). One SAE occurred during the study, a cellulitis of the upper leg requiring two days of hospitalization. The SAE was considered unlikely to be related to the study drug as no skin lesions on the upper leg were included for the respective patient. Severity of all AEs (n = 45) was classified as mild (n = 42) or moderate (n = 3) and all resolved without sequelae. No discontinuations due to AEs or deaths occurred in both parts.

Topical application of bimiralisib did not result in any clinically significant changes in vital signs, laboratory analyses, ECG or urinalysis for both parts. LIGS scores in part A were comparable between the bimiralisib and placebo treatment areas during the full course of the study part (Table S5). In part B, two patients (1/10 in placebo group and 1/9 in bimiralisib group) experienced mild dryness at the application site on day 14, and one patient experienced well-defined erythema at the application site in the bimiralisib group on day 28 (Table S6).

### 3.3. Clinical Efficacy and Pharmacodynamics

**Figure 3:**
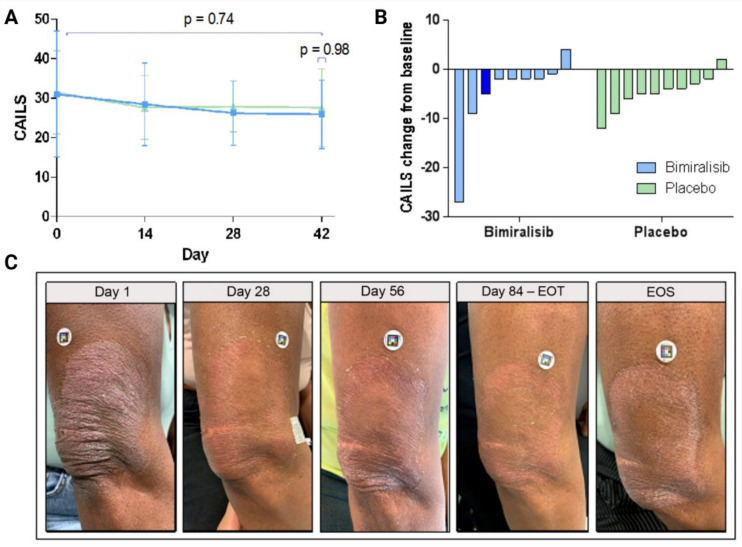
Clinical efficacy of bimiralisib 2.0% gel compared to placebo, by lesion improvement measured by CAILS in the per protocol population. (**A**) Absolute total CAILS score on days 14, 28 and 42 of daily topical gel application. No statistical significant difference in efficacy was detected between bimiralisib and placebo over time (*p* = 0.74) or on day 42 (*p* = 0.98). (**B**) Individual change of CAILS from baseline per patient. The dark blue bar indicates the patient shown in Figure 3C. (**C**) Clinical response of a single early-stage MF patient by Scarlet Red clinical pictures.

Upon 42 days of treatment, no statistically significant changes from baseline in CAILS were observed between bimiralisib and placebo (0.0; 95% CI, −3.3–3.4, p = 0.9820) (Figure 3A/B).

No substantial differences were detected in objective response rates between treatment groups (Table S7). Clinically, all subjects were characterized by stable disease, except for one subject (10%) on placebo who experienced disease progression. Three subjects in the bimiralisib group showed ≥15% lesional response by CAILS and continued into the blinded extension period; Figure 3C shows the clinical response over time for a single patient. Target lesion assessment of skin perfusion (LSCI), blood flow (OCT) and erythema and roughness by 3D multispectral imaging showed no improvement in bimiralisib- or placebo-treated patients (Table S8).

### 3.4. Systemic and Cutaneous Pharmacokinetics

**Figure 4:**
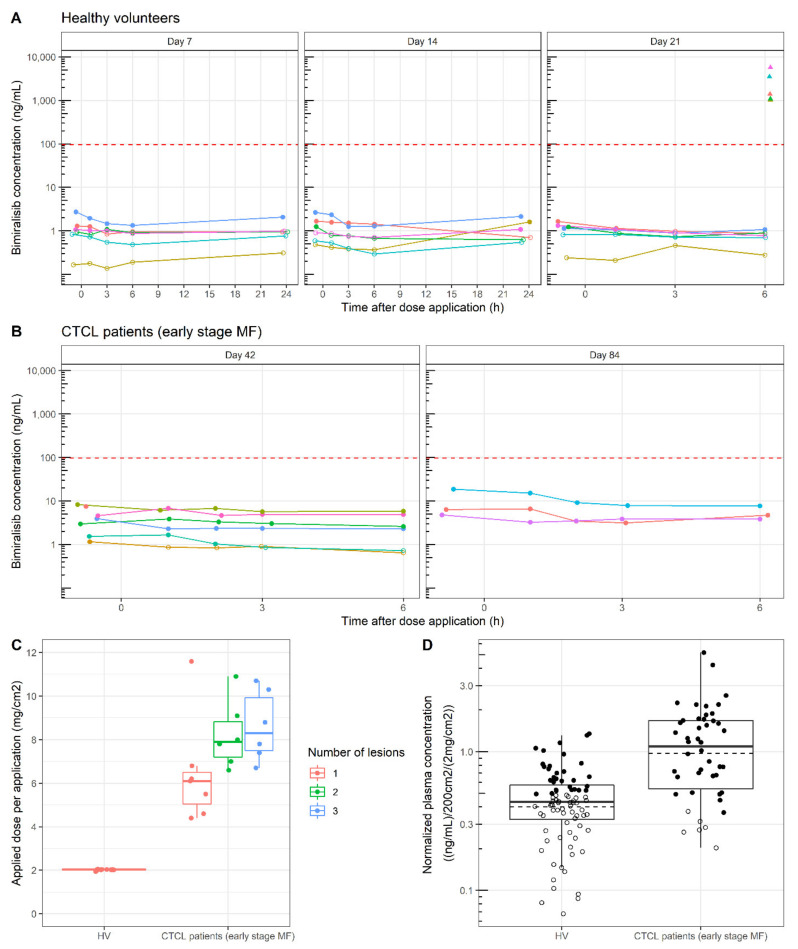
Bimiralisib pharmacokinetics in HVs and early-stage MF patients. (**A**) Individual plasma concentrations of 6 healthy volunteers after 7, 14 and 21 days of topical daily application ranged from 0.14 to 2.74 ng/mL. The red dotted line is the NOAEL Cmax (96.6 ng/mL). (**B**) Individual plasma concentrations of 9 MF patients, 6 patients after 42 days of daily topical application and 3 patients after 84 days of bimiralisib application. Concentrations ranged from 0.67 to 18.70 ng/mL. (**C**) Applied dose per application increased with increasing target lesions, as shown by exploratory analysis. Patients with a single target lesion applied less gel per dose (6.5 ± 2.4) in comparison to patients with two (8.2 ± 1.6) or three (8.6 ± 1.6) target lesions. (**D**) Normalized plasma concentration was 2.44-fold higher for patients compared to healthy volunteers. Boxplots show the first, median and third quartiles in the box; whiskers extend up to 1.5× the interquartile range. Dashed line is the log-transformed mean. Open dots are data below the LLOQ (1.0 ng/mL).

For HVs, bimiralisib concentrations in plasma were detectable but low and substantially below the human no observed adverse effect level (NOAEL, 96.6 ng/mL, [26]) (Figure 4A, NOAEL indicated by dashed red line).

The highest C_max_ was 2.74 ng/mL and the C_avg_ for all HVs was 0.956 ng/mL. After 14 days of once-daily bimiralisib gel administration, the highest AUC_(0-24h)_ was 40 h·ng/mL, and the mean AUC_(0–24 h)_ on day 7 of 21.3 h·ng/mL was comparable to the AUC_(0–24 h)_ on day 14 of 22.2 h·ng/mL, suggesting that steady state of topical bimiralisib gel is reached before day 7 and exposure in a 24 h interval does not increase from day 7 onwards.

Bimiralisib concentrations measured in plasma were higher for MF patients than for HVs (highest concentration reached was 18.70 ng/mL) but still substantially below the NOAEL (Figure 4B). The C_avg_ for all MF patients was increased to 4.49 ng/mL. However, the increased exposure in MF patients should be corrected for changes in the applied dose and for the treated lesion area as Figure 4C clearly shows that the applied dose in HVs was well controlled in a clinical setting, compared to the applied dose in MF patients in an at-home setting. An increase in the number of treated lesions showed a positive correlation with the applied dose, and a mean (± SD) dose of up to 8.1 mg/cm^2^ (± 2.6) mg/cm^2^ bimiralisib was applied compared to the target dose of 2 mg/cm^2^. Patients with a single target lesion applied less gel per dose (6.5 ± 2.4 mg/cm^2^) in comparison to patients with two (8.2 ± 1.6 mg/cm^2^) or three (8.6 ± 1.6 mg/cm^2^) target lesions. The mean ratio of the normalized concentration (normalized around 200 cm^2^/(2 mg/cm^2^)) was 2.44, indicating a 144% increase in concentration for MF patients compared to HVs after identical treatments (Figure 4D). This observation means that there was an increased penetration of bimiralisib in MF patients.

Bimiralisib exposure levels measured in skin punch biopsies of HVs showed a mean concentration of 2.54 µg/g, ranging between 1.03 and 5.7 µg/g. The mean ratio for skin/plasma concentration of bimiralisib in HVs was 3052, ranging between 1212 and 7360 times higher.

**Figure 5:**
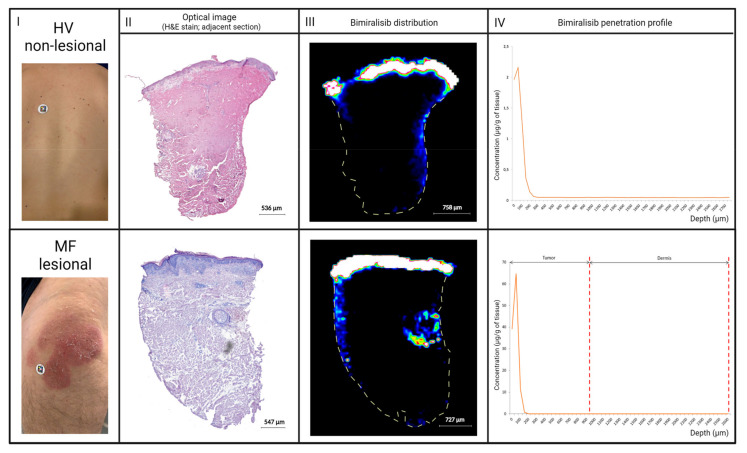
Individual cutaneous pharmacokinetics of a single HV compared to a single early-stage MF patient. Left to right: (I) Clinical picture. (II) H&E slide visualizing epidermis and dermis. (III) Bimiralisib distribution by MALDI image. Distribution in the lateral sides of the dermis of both biopsies is considered punch contamination. (IV) Penetration profile of areas selected without contamination.

The mean exposure levels in MF patients were higher (5.3 µg/g, 0.3–30.2 µg/g) compared to HVs but should be seen in light of the higher dose applied to the lesion area. Exposure in the papillary dermis was approximately 10-fold lower, as is visible in Figure 5 comparing skin punch biopsies of a single HV and MF patient and cutaneous spatial pharmacokinetics by MALDI-MSI (Figure 1).

## 4. Discussion

### 4.1. Topical Bimiralisib Is Safe and Has a Favorable Pharmacokinetic Profile

In this first-in-human study, we investigated the safety, efficacy and pharmacokinetics of topical bimiralisib in healthy volunteers and MF patients. Importantly, we show a clear cutaneous PK profile for MF patients with a topical treatment for the first time. Of note, this clinical trial shows the use of bimiralisib in gel formulation is safe and well tolerated. There were no serious adverse events related to bimiralisib, and treatment-emergent adverse events were mostly mild or a few moderate, all transient, fully reversible and self-limiting. This is a major improvement compared to the severe adverse events that were seen with oral and intravenous administration of bimiralisib [11,16,17,18,19,20].

Systemic pharmacokinetics investigations detected concentrations that were substantially below the NOAEL in both HVs and patients. After correction for lesion area and applied dose, the systemic exposure to bimiralisib was higher in MF patients compared to HVs. A possible explanation might be lesional vasodilation [27]. Furthermore, CTCL lesions show decreased filaggrin and skin moisture levels [28], i.e., inducing desquamation and a skin barrier dysfunction [29]. Additionally, this is comparable to the nearly 2-fold increased skin absorption found in patients with atopic dermatitis when compared to HVs [30]. Skin barrier impairment in both diseases indicates a higher risk of systemic exposure to topical products. Local bimiralisib exposure levels measured in skin punch biopsies of part A were within range of the IC90 of pAKT and pS6 required to inhibit the mTOR pathway in the epidermis [31,32], but bimiralisib exposure was approximately 10-fold lower in the papillary and reticular dermis. This could as well be explained by the hypothesized rapid drug absorption due to an increase in blood flow following vasodilation, increasing vascular permeability [33,34]. Of note, strengthening the higher exposure to bimiralisib seen in MF patients, the observed high body weight of the included MF patients compared to HVs contributes to a higher volume of distribution in patients, suggesting exposure in patients was expected to be lower than that in HVs.

### 4.2. Potential Reasons for Lack of Efficacy—A Question-Based Approach

Although topical bimiralisib has a favorable pharmacokinetic and safety profile, no statistically significant clinical response of the early-stage MF was observed over six weeks of daily topical application. Multiple reasons for the lacking efficacy can be hypothesized.

The first question that arises is whether topical bimiralisib reaches the site of action. Malignant T cells in early-stage MF typically reside in the epidermis and papillary dermis [35,36,37]. From cutaneous PK data in HVs and MF patients and the systemic PK exposure, we can conclude that the skin barrier, i.e., stratum corneum, has been overcome by bimiralisib. Therefore, it is certain that bimiralisib reaches the papillary dermis in patients. Presumably, the vasodilation seen in most MF lesions [27] may have caused a more rapid uptake in systemic circulation. However, no direct proof is available that there was insufficient exposure of tumor cells to the drug or drug uptake into the target cells.

Furthermore, in case bimiralisib reaches the site of action and can penetrate the malignant T cells, it is questionable whether the time of drug residence is adequate to facilitate target engagement. Active MF lesions are by definition erythematous, imputable to vasodilation [27] resulting in higher blood perfusion. The increased blood perfusion and vasodilation in the dermis might lead to a (too) short time of drug residence for target engagement, which raises the second question: does topical bimiralisib cause its intended pharmacological effect? However, we did not investigate target engagement by analysis of pS6, a downstream effector of the PI3K/AKT/mTOR pathway, directly, leaving this question unanswered.

The third question that arises is the relevance of the mechanism of action of bimiralisib in (part of the) MF patients. Crosstalk in signaling pathways and compensatory pathways exerting the same basal cell cycle and survival functions, such NF-κB pathways [38] and MAPK/ERK kinase [39], can occur when inhibiting the PI3K/AKT/mTOR pathway. Combination therapy may overcome this crosstalk activation of tumor pivotal pathways [40,41]. However, dual pan-class PI3K/mTOR inhibitors were developed to overcome this multiple pathway issue and seem to do so in preclinical evidence [42], showing in vitro and in vivo preclinical antitumor activity in lymphomas as monotherapy and combination therapy [43]. Notwithstanding, even with dual inhibition of pan-class PI3K and both mTOR complexes, it can be hypothesized that, e.g., TCR/PLCγ1-NFAT, MAPK/ERK, TNFR-NF-κB and JAK/STAT signaling pathways, important in pathogenesis and progression of MF [44,45,46,47,48,49,50,51,52,53,54], could undergo upregulation upon full inhibition of the PI3K/AKT/mTOR pathway. In addition, single-cell genetic analyses revealed marked inter- and intratumoral heterogeneity in MF patients [55,56]. The PI3K/AKT/mTOR pathway may thus have had a marginal role, if a role at all, in the individual pathogenesis of mycosis fungoides in the included early-stage patients.

### 4.3. CAILS: Subjected to Subjectivity?

The main efficacy outcome for this clinical trial was CAILS. Although CAILS is the most commonly used method for MF lesion severity scoring [22], CAILS has only one in five quantitative measures and is thus prone to subjectivity and inter- and intraindividual variability, as is well known for similar dermatological visually scored severity scores [57,58,59]. The variability in scoring is fueled by the lack of existing consensus definitions in severity scoring per subjective point on the eight-point scale. For example, a component of CAILS is scoring hyper- and/or hypopigmentation. Degree of hypopigmentation is more difficult to visualize with decreasing Fitzpatrick skin type. Subsequently, hyperpigmentation can be observed in 58.6% of progressing lesions in CD8+ cytotoxic mycosis fungoides [60] but can also occur after inflammation indicating lesion improvement in classical MF. In this regard, lesion improvement showing a marked decrease in erythema, but with associated post-inflammatory hyperpigmentation, will not facilitate an improvement in CAILS. This study aimed to overcome this difficulty in subjective scoring by successfully implementing objective measuring methods to map MF lesions, supporting the clinical assessment of the pharmacodynamic effect of topical bimiralisib.

In conclusion, we showed that bimiralisib gel 2% for topical use leads to (I) meaningful cutaneous drug levels, (II) well-tolerated systemic drug exposure in patients with MF and (III) a lack of clinical efficacy. The last was subject to numerous unknown factors, in need of further exploration before bimiralisib in topical formulation is depreciated as a novel therapeutic drug for primary cutaneous lymphomas.

## Figures and Tables

**Table 1:** Study design of part A with HVs (n = 6) and part B with MF patients (n = 19). Patients could continue in an extended treatment period if they had ≥15% lesion improvement by CAILS on day 42.

Part A	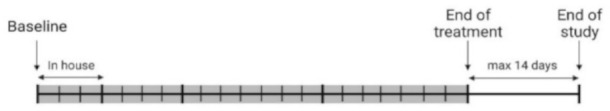								
Day	0–4	5–6	7	8–13	14	15–20		21	35
Outpatient visit	X	X	X	X	X	X		X	X
Vital signs	X	X	X	X	X	X		X	X
Safety assessments	X		X		X			X	X
Systemic PK assessment	X		X		X			X	
Biopsy								X	
**Part B**	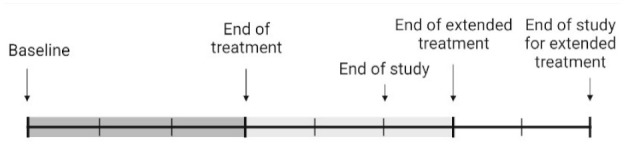								
Day	0	14	28	42	56	70	84	98	112
Outpatient visit	X	X	X	X	X	X	X	X	X
Safety assessments	X	X	X	X	X	X	X		X
Efficacy assessment	X	X	X	X	X	X	X	X	X
Systemic PK assessment	X			X			X		
Biopsy				X			X		

**Table 2:** Baseline and disease characteristics of HVs and MF patients.

	HV (n = 6)	MF Bimiralisib 2% (n = 9)	MF Vehicle Gel (n = 10)
Age, years (SD, range)	31.7 (13.3, 21–50)	52.0 (12.2, 31–70)	55.2 (16.4, 21–78)
Sex, n (%)			
Female	0	4 (44.4)	3 (30.0)
Male	6 (100)	5 (55.6)	7 (70.0)
Weight, kg (SD, range)	80.8 (13.3, 61.2–94.7)	94.5 (9.3, 79.4–109.2)	93.24 (16.2, 71.5–124.8)
BMI, kg/m^2^ (SD, range)	23.7 (2.5, 20.7–27.4)	29.9 (3.0, 26.7–35.7)	29.6 (4.6, 25.0–39.2)
Fitzpatrick skin type (%)			
I	0	0	1 (10.0)
II	2 (33.3)	3 (33.3)	5 (50.0)
III	3 (50)	4 (44.4)	3 (30.0)
IV	1 (16.7)	0	1 (10.0)
V	0	1 (11.1)	0
VI	0	1 (11.1)	0
Lesion, n (%)			
1		3 (33.3)	4 (40.0)
2		4 (44.4)	2 (20.0)
3		2 (22.2)	4 (40.0)
mSWAT (SD)			
Patch sum (%BSA*1)		11.4 (7.3)	16.2 (9.7)
Plaque (%BSA*2)		4.0 (9.2)	3.0 (4.9)
Total mSWAT score		15.4 (10.6)	19.2 (12.8)
Treatment history, n (%)			
Topical corticosteroids		9 (100.0)	9 (90.0)
UVB		0	1 (10.0)
PUVA		5 (55.6)	3 (30.0)
Interferon-α		1 (11.1)	0
Radiotherapy		0	1 (10.0)

## Data Availability

Restrictions apply to the availability of these data. Data are owned by Swissrockets and are available with the permission of Swissrockets.

## Supplementary Materials

The following are available online at https://www.mdpi.com/article/10.3390/cancers14061510/s1, Table S1: All eligibility criteria for HVs and MF patients, Table S2: Sample size, randomization and stopping criteria, Figure S1: Study flow chart of the clinical safety run-in (part A) with HVs, Table S3: TEAEs in HVs, Table S4: TEAEs in MF patients, Table S5: LIGS for HVs, Table S6: LIGS for MF patients, Table S7: ORR over time on treatment for MF patients. Table S8: Results of pharmacodynamic assessments for MF patients.
