# Correction: Wind et al. Topical Bimiralisib Shows Meaningful Cutaneous Drug Levels in Healthy Volunteers and Mycosis Fungoides Patients but No Clinical Activity in a First-in-Human, Randomized Controlled Trial. *Cancers* 2022, *14*, 1510

**DOI:** 10.3390/cancers15051485

**Published:** 2023-02-27

**Authors:** Selinde S. Wind, Manon A. A. Jansen, Melanie Rijsbergen, Michiel J. van Esdonk, Dimitrios Ziagkos, Wing C. Cheng, Tessa Niemeyer-van der Kolk, John Korsten, Agnieszka Gruszka, Debora Schmitz-Rohmer, David Bonnel, Raphael Legouffe, Florian Barré, Marcel W. Bekkenk, Ellen R. M. de Haas, Koen D. Quint, Harald Schnidar, Melanie Rolli, Henk Johan Streefkerk, Jacobus Burggraaf, Maarten H. Vermeer, Robert Rissmann

**Affiliations:** 1 Centre for Human Drug Research, 2333 CL Leiden, The Netherlands; 2 Department of Dermatology, Leiden University Medical Center, 2333 ZA Leiden, The Netherlands; 3 Charles River Laboratories Den Bosch B.V., 5231 DD Den Bosch, The Netherlands; 4 PIQUR Therapeutics AG, 4057 Basel, Switzerland; 5 MS Imaging Department, ImaBiotech, 59120 Lille, France; 6 Amsterdam University Medical Centers, 1105 AZ Amsterdam, The Netherlands; 7 Erasmus Medical Center, 3015 GD Rotterdam, The Netherlands; 8 SCARLETRED Holding GmbH, 1030 Vienna, Austria; 9 Leiden Academic Centre for Drug Research, Leiden University, 2333 CC Leiden, The Netherlands

The authors wish to make the following corrections to this paper [1]: 

1. ‘Harald Schnidar’ was not included as an author in the published article and he was added as the co-author including his affiliation 8. The corrected Author Contributions Statement was updated as follows:

The author contributed to this research in the development, supply and read-out of software for this clinical trial. His contribution was added to ‘Author Contributions’ sections: ‘conceptualization’, ‘software’, ‘writing—review and editing’ and ‘visualization’ of the manuscript.

2. The medical device product name and the regarding sentence were changed to ‘For standardized daily photo documentation and objective erythema quantification of a single target lesion, the CE medical device certified mobile app Scarletred^®^Vision (SCARLETRED, Vienna, Austria) [23,24] was used by patients at home and investigators on site’ in Section 2.3. 

3. The caption of Figure 3C was updated to ‘SCARLETRED imaging’. For Figure 5I caption, ‘by SCARLETRED imaging’ was added and the statement was corrected to: ‘(I) Clinical picture by SCARLETRED imaging’. 

4. Furthermore, a new reference [2] was added as 23th reference for completeness regarding the objective quantification of erythema by SCARLETRED imaging. All subsequent after reference [23] were numbered sequentially. 

Finally, to be consistent with the cited references, the ‘intravenous’ administration of bimiralisib was removed from the Introduction on page 2 and Section 4.1 of the Discussion on page 12. The corrected sentences reads now: ‘Multiple clinical studies, including studies on 233 patients with various malignancies (including lymphomas), demonstrated potential clinical activity of oral bimiralisib’ and ‘This is a major improvement compared to the severe adverse events that were seen with oral administration of bimiralisib [11,16–20].’ respectively.

The authors apologize for any inconvenience caused and state that the scientific conclusions are unaffected. The original article has been updated. The original version will remain online on the article webpage, with a reference to this erratum. 
