# Supplementary material for: Topical Bimiralisib Shows Meaningful Cutaneous Drug Levels in Healthy Volunteers and Mycosis Fungoides Patients but No Clinical Activity in a First-in-Human, Randomized Controlled Trial
Source: Cancers (Basel). 2022 Mar 15;14(6):1510. doi: 10.3390/cancers14061510 (PMC8946662; doi:10.3390/cancers14061510)
Supplement: Supplementary file 1 [file cancers-14-01510-s001.zip › cancers-1537602 supplementary.pdf]

Supplementary Material

Table S1. In- and exclusion criteria.

**Inclusion criteria**
**Healthy volunteers may be included in the study if they meet all the below inclusion criteria:**<br>1. Males ≥ 18 years of age;<br>2. Fitzpatrick skin type I, II, III or IV;<br>3. No clinically significant skin disease in the treatment area(s) and no history of hypertrophic Scarring;<br>4. Willing and able to washout and withhold any topical treatment in the treatment area (2 weeks);<br>5. Subjects must understand the investigational nature of this study and sign an independent ethics committee-approved written informed consent form prior to any study related procedure;<br>6. Willing to comply with all study requirements;<br>7. Subjects of reproductive potential must agree to use double contraception from screening until 90 days after discontinuing study treatment and withhold from any sperm donation.
**Patients may be included in the study if they meet all the below inclusion criteria:**<br>1. Males or females ≥ 18 years of age;<br>2. Confirmed diagnosis of CTCL (MF): MF stage 1A or 1B (maximum T2N0M0B1) as per Modified ISCL/EORTC revisions (Olsen-2011): Confirmed histopathological diagnosis from skin biopsy representative of current disease by pathologist with expertise in cutaneous lymphoma. The date of biopsy should be within the last 5 years. If diagnosis is not confirmed by light microscopic examination, ISCL diagnostic criteria must be used;<br>3. At least 1, 2, or 3 target lesions with a total (combined) size of at least 150 cm^2;<br>4. Willing and able to washout any previous topical treatment (at least 2 weeks) and any systemic treatment (at least 4 weeks) prior to first application of topical bimiralisib;<br>5. Otherwise healthy, i.e. absence of clinically significant or unstable disease, with acceptable organ function with lab values within normal range or as specified below:<br>a. eGFR (mCockcroft-Gault) > 30 mL/min<br>b. AST and ALT <= 2.5x ULN<br>c. Total bilirubin <= 1.5x ULN (except patients with Gilbert's syndrome, who may have total bilirubin <= 3x ULN)

d. Platelet count >= 100.000 $/mm^3$
e[1]. WBC count >= 3000 $/mm^3$
f[1]. ANC count >= 3000 $/mm^3$
g[2]. Fasting blood glucose <= 125 mg/dL
6. Patient must understand the investigational nature of this study and sign an independent ethics committee/ approved written informed consent form prior to any study related procedure;
7. Willing to comply with all study requirements;
8. Patients of reproductive potential must agree to use double effective contraception from screening until 90 days after discontinuing study treatment;
9. Females who had a menstrual cycle within 2 years prior to Screening must have a negative serum pregnancy test at Screening and a negative urine pregnancy test on their first dosing day.

[1]Eligibility criteria for white blood cell count and absolute neutrophil count were widened to >= 1500 $/mm^3$ after trial commencement to enable more patients to participate in the study, following data available from the healthy volunteer cohort indicating systemic exposure was much lower than would be expected to cause systemic adverse events.
[2]Fasting blood glucose was removed from the inclusion criteria after trial commencement, due to low probability for systemic adverse events and high burden for many MF patients, of whom some were known with a well-controlled diabetes mellitus type II.

Exclusion criteria

Healthy volunteers will be excluded from the study if they meet any of the below exclusion criteria:
1. Known hypersensitivity to any of the excipients of bimiralisib gel.
2. Evidence of any clinically significant active or unstable chronic disease or condition that could interfere with, or for which the treatment of might interfere with, the conduct of the study, or that would pose an unacceptable risk to the subject in the opinion of the investigator (following a detailed medical history, physical examination, vital signs (systolic and diastolic blood pressure, pulse rate, body temperature) and 12-lead electrocardiogram (ECG) at screening or pre-dose). Minor deviations from the normal range may be accepted, if judged by the Investigator to have no clinical relevance.
3. Clinically significant abnormalities, as judged by the investigator, in laboratory test results (including hepatic and renal panels, complete blood count, chemistry panel and urinalysis) at screening or pre-dose. In the case of uncertain or questionable results, tests performed during screening may be repeated before randomisation to confirm eligibility or judged to be clinically irrelevant for healthy subjects.

4. Positive Hepatitis B surface antigen (HBsAg), Hepatitis C antibody (HCV Ab), or human immunodeficiency virus antibody (HIV Ab) at screening, or other known infection requiring antibiotic therapy within the last three months prior to the study.
5. Participation in an investigational drug or device study within 3 months prior to screening or more than 4 times in the past year.
6. Donation of blood or blood loss of >500 mL within 3 months prior to screening or donation of plasma within 14 days.
7. Any condition that in the opinion of the investigator would complicate or compromise the study or the well-being of the subject.

Patients will be excluded from the study if they meet any of the below exclusion criteria:
1. Known hypersensitivity to any of the excipients of bimiralisib gel.
2. Patients who are on (or will require) any systemic treatment to treat their disease (MF) during the study.
3. Concurrent severe and/or uncontrolled medical conditions that would, in the investigator's judgment, contra-indicate patient participation in the clinical study or require concomitant skin-directed or systemic therapy (e.g., active or uncontrolled severe infection, chronic active hepatitis, immuno-compromised, acute or chronic pancreatitis, uncontrolled high blood pressure, interstitial lung disease, etc.).
4. Has other active malignancies that require systemic treatment.
5. Has a known history of HIV infection or hepatitis (testing not mandatory)
6. Pregnant or nursing (lactating) women.
7. Has a known history of alcohol or drug abuse within the past 1 year.
8. Psychiatric illness, disability or social situation that would compromise the subject's safety or ability to provide consent, or limit compliance with study requirements.

Table S2. Sample size calculation, randomisation and stopping criteria.

**Sample size calculation**
As this is the first clinical study where topical bimiralisib was applied to humans in general and CTCL patients in particular, the standard deviation (SD) of the primary endpoint in part B - the mean percentage change from baseline on CAILS - had to be approximated from data available in relevant literature. Rook et al described the results of treatment with various doses of topical resiquimod gel for eight weeks in 12 early stage CTCL patients, showing a mean percentage change from baseline on CAILS of 75% with an estimated between subject SD of 27.1% [25]. In another study, oral duvelisib (a PI3K inhibitor) was observed to have a 31.6% response rate (ORR)

in 19 CTCL patients [15]. For the power calculation of the present study, therefore a common SD of 30% is chosen, with an effect of 51% for bimiralisib. In order to detect between-treatment difference of 42% with a common SD of 30%, a two sided alpha of 0.05 and a power of 80%, assuming the vehicle group shows a mean effect of 9% and the bimiralisib group shows an effect of 51%, at least 9 patients in each treatment arm were to be included. Using a randomisation ratio of 1:1, inclusion of 18 MF patients for part B was planned.

Randomisation

Subjects were randomised in a consecutive order starting with the lowest number. The SAS code for the parallel, two treatment randomisation with ratio 1:1 was generated by a study-independent statistician, with block size 2. The randomisation code was unblinded and made available for data analysis directly after the last evaluable patient has had his/her 6wk efficacy assessment, i.e., when collection of the primary endpoint data has been completed, the protocol deviations determined, and the clinical database declared complete, accurate and locked for all information pertaining to the primary endpoint. The randomisation code was kept strictly confidential. Sealed individual randomisation codes, per subject and per treatment, were placed in a sealed envelope, labelled 'emergency decoding envelopes', which was kept in a safe cabinet at the study site. The study-independent statistician shared with the bioanalytical expert which samples had to be analysed. The bioanalytical expert provided blinded results back to the study-independent statistician, to support the safety review. The bioanalytical expert was not otherwise involved in the study.

Stopping criteria

Application of bimiralisib or vehicle had to be stopped in case of an unacceptable tolerability profile based on the nature, frequency, and intensity of observed AEs judged jointly by the investigator and the sponsor.

Application of bimiralisib or vehicle had to be (temporarily) interrupted if any of the following dose limiting toxicities (DLTs) occurred:
Any Erythema Grade 4 or higher
Any SAE suspected related to study drug by the investigator, or
Any Grade 4 or higher (as per CTCAE criteria v5.0) AE suspected related to study drug by the investigator.

If, during the treatment of healthy volunteers, no DLTs were observed, but 4 or more participants experienced a Grade 3 event related to IMP, the dosing of the complete cohort had to be stopped. Similarly, if one participant experienced 1 or more DLTs, and two or more other participants

experienced one or more Grade 3 events related to IMP, the dosing of the complete cohort had to be stopped. Finally, if two or more participants experienced 1 or more DLTs, the dosing of the complete cohort had to be stopped.

Figure S1. Study flow chart of the clinical safety run-in (part A) with healthy volunteers. Eleven subjects were excluded due to the following: not meeting inclusion criteria (n=5), withdrew from participation (n=3) and study was full (n=3).

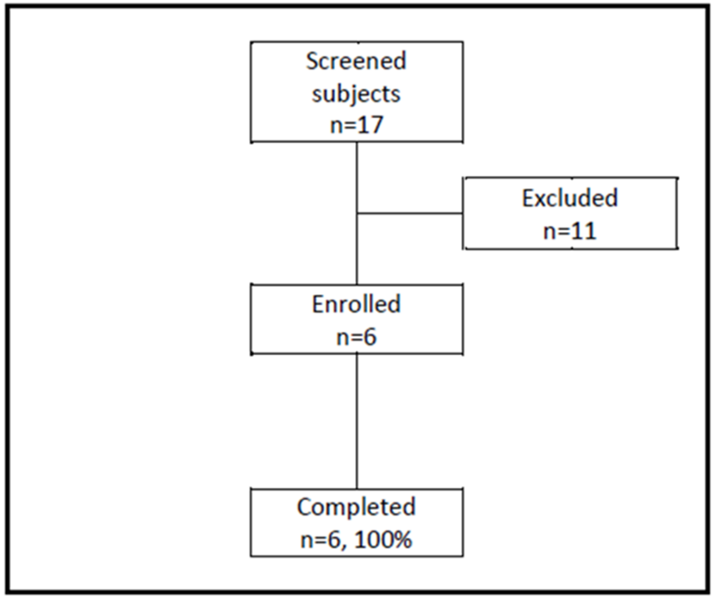

Table S3. Treatment Emergent Adverse Events in HV (part A).

System Organ Class/Preferred Term	Events N	Subjects N (%)
**Any events**	20	6 (100.0)
**General disorders and administration site conditions**	10	5 (83.3)
Application site acne	3	3 (50.0)
Application site discomfort	1	1 (16.7)
Application site dryness	5	5 (83.3)
Application site pruritus	1	1 (16.7)
Application site pustules	1	1 (16.7)
Application site erythema	2	2 (33.3)
**Infections and infestations**	1	1 (16.7)
Influenza	1	1 (16.7)
**Musculoskeletal and connective tissue disorders**	2	2 (33.3)
Back pain	2	2 (33.3)
**Nervous system disorders**	4	2 (33.3)
Dysesthesia	1	1 (16.7)
Headache	2	1 (16.7)
Tremor	1	1 (16.7)
**Skin and subcutaneous tissue disorders**	3	3 (50.0)
Application site acne	1	1 (16.7)
Erythema	2	2 (33.3)

Table S4. Treatment Emergent Adverse Events in MF patients (part B).

	**Part B – MF patients**	
**System Organ Class/Preferred Term**	**Bimiralisib (n=9), Subjects (%)**	**Placebo (n=6), Subjects (%)**
**Total reported adverse events (% subjects)**	22 (66.7)	23 (90.0)
**Cardiac and vascular disorders**	1 (11.1)	2 (10.0)
Aortic valve sclerosis	0	1 (10.0)
Mitral valve incompetence	0	1 (10.0)

Hypertension	1 (11.1)	0
**General disorders, administration site conditions and skin and subcutaneous tissue disorders**	4 (33.3)	7 (40.0)
Application site pain	0	1 (10.0)
Application site paresthesia	0	1 (10.0)
Application site pruritus	1 (11.1)	1 (10.0)
Application site warmth	0	1 (10.0)
Burning sensation	1 (11.1)	0
Pruritus	1 (11.1)	2 (20.0)
Pain	0	1 (10.0)
Peripheral coldness	1 (11.1)	0
**Ear and labyrinth disorders**	0	1 (10.0)
Otitis externa	0	1 (10.0)
**Endocrine disorders**	0	1 (10.0)
Hypoglycemia	0	1 (10.0)
**Infections and infestations**	5 (22.2)	1 (10.0)
Cellulitis	3 (11.1)	0
Rhinitis	1 (11.1)	0
Sebaceous gland infection	1 (11.1)	1 (10.0)
**Injury, poisoning and procedural complications**	1 (11.1)	0
Tooth fracture	1 (11.1)	0
**Metabolism and nutrition disorders**	1 (11.1)	0
Vitamin D deficiency	1 (11.1)	0
**Neoplasms benign, malignant and unspecified**	0	3 (30.0)
Mycosis fungoides stage IB	0	1 (10.0)
Mycosis fungoides stage IIB	0	2 (20.0)

**Musculoskeletal and connective tissue disorders**	4 (33.1)	2 (20.0)
Arthralgia	0	1 (10.0)
Back pain	1 (11.1)	0
Musculoskeletal stiffness	1 (11.1)	1 (10.0)
Myalgia	2 (22.2)	0
**Nervous system disorders**	3 (22.2)	5 (30.0)
Headache	1 (11.1)	5 (30.0)
Migraine without aura	1 (11.1)	0
Presyncope	1 (11.1)	0
**Respiratory, thoracic and mediastinal disorders**	3 (22.2)	1 (10.0)
Cough	1 (11.1)	0
Rhinorrhea	1 (11.1)	1 (10.0)
Viral sinusitis	1 (11.1)	0

Table S5. A: Local Irritation Grading Scale for HV in part A – area 1 (placebo). Desquamation: CAT1 = none, CAT2 = dryness, CAT3 = thin scales, CAT4 = moderate scales, CAT5 = Large scales. Edema: CAT1 = none, CAT2 = very mild, CAT3 = mild, CAT4 = moderate, CAT5 = intense. Erythema: CAT1 = none, CAT2 = very mild, CAT3 = well-defined, CAT4 = moderate, CAT5 = severe. Overall: CAT1 = No reaction/doubtful, CAT2 = mild reaction, CAT3 = moderate reaction, CAT4 = intense reaction.

**LIGS placebo area**	**CAT1 N (%)**	**CAT2 N (%)**	**CAT3 N (%)**	**CAT4 N (%)**	**CAT5 N (%)**
Highest on treatment - Desquamation	1 (16.7)	2 (33.3)	3 (50.0)	0	0
Highest on treatment - Edema	6 (100.0)	0	0	0	0
Highest on treatment - Erythema	3 (50.0)	3 (50.0)	0	0	0
Highest on treatment – Overall	5 (83.3)	1 (16.7)	0	0	-
Highest post-treatment - Desquamation	6 (100)	0	0	0	0
Highest post- treatment - Edema	6 (100)	0	0	0	0
Highest post-treatment - Erythema	6 (100)	0	0	0	0
Highest post-treatment - Overall	6 (100)	0	0	0	-

Table S5. B: Local Irritation Grading Scale for HV in part A – area 2 (bimiralisib).

LIGS bimiralisib area	CAT1 N (%)	CAT2 N (%)	CAT3 N (%)	CAT4 N (%)	CAT5 N (%)
**Highest on treatment - Desquamation**	2 (33.3)	3 (50.0)	1 (16.7)	0	0
Highest on treatment - Edema	6 (100.0)	0	0	0	0
Highest on treatment - Erythema	3 (50.0)	2 (33.3)	1 (16.7)	0	0
Highest on treatment – Overall	6 (100.0)	0	0	0	-
Highest post-treatment - Desquamation	6 (100)	0	0	0	0
Highest post- treatment - Edema	6 (100)	0	0	0	0
Highest post-treatment - Erythema	6 (100)	0	0	0	0
Highest post-treatment - Overall	6 (100)	0	0	0	-

Table S6. Local Irritation Grading Scale for MF patients for bimiralisib and placebo group. Application site reactions were scored as positive if reaction was visible outside of target lesion (except for edema). Desquamation: CAT1 = none, CAT2 = dryness, CAT3 = thin scales, CAT4 = moderate scales, CAT5 = Large scales. Edema: CAT1 = none, CAT2 = very mild, CAT3 = mild, CAT4 = moderate, CAT5 = intense. Erythema: CAT1 = none, CAT2 = very mild, CAT3 = well-defined, CAT4 = moderate, CAT5 = severe. Overall: CAT1 = No reaction/doubtful, CAT2 = mild reaction, CAT3 = moderate reaction, CAT4 = intense reaction.

LIGS	Bimiralisib					Placebo				
	CAT1 N (%)	CAT2 N (%)	CAT3 N (%)	CAT4 N (%)	CAT5 N (%)	CAT1 N (%)	CAT2 N (%)	CAT3 N (%)	CAT4 N (%)	CAT5 N (%)
Day 14 - Desquamation	8 (88.9)	1 (11.1)	0	0	0	9 (90)	1 (10)	0	0	0
Day 14 - Edema	9 (100)	0	0	0	0	10 (100)	0	0	0	0
Day 14 - Erythema	9 (100)	0	0	0	0	10 (100)	0	0	0	0
Day 14 – Overall	9 (100)	0	0	0	0	10 (100)	0	0	0	0
Day 28 - Desquamation	9 (100)	0	0	0	0	9 (90)	0	0	0	0
Day 28 - Edema	9 (100)	0	0	0	0	9 (90)	0	0	0	0
Day 28 - Erythema	8 (88.9)	0	1 (11.1)	0	0	9 (90)	0	0	0	0
Day 28 - Overall	9 (100)	0	0	0	0	9 (90)	0	0	0	0
Day 42 - Desquamation	9 (100)	0	0	0	0	9 (90)	0	0	0	0
Day 42 - Edema	9 (100)	0	0	0	0	9 (90)	0	0	0	0
Day 42 - Erythema	9 (100)	0	0	0	0	9 (90)	0	0	0	0
Day 42 – Overall	9 (100)	0	0	0	0	9 (90)	0	0	0	0
Day 56 - Desquamation	3 (33.3)	0	0	0	0	2 (20)	0	0	0	0
Day 56 - Edema	3 (33.3)	0	0	0	0	2 (20)	0	0	0	0
Day 56 - Erythema	3 (33.3)	0	0	0	0	2 (20)	0	0	0	0

Day 56 – Overall	3 (33.3)	0	0	0	0	2 (20)	0	0	0	0
Day 70 - Desquamation	3 (33.3)	0	0	0	0	2 (20)	0	0	0	0
Day 70 - Edema	3 (33.3)	0	0	0	0	2 (20)	0	0	0	0
Day 70 - Erythema	3 (33.3)	0	0	0	0	2 (20)	0	0	0	0
Day 70 – Overall	3 (33.3)	0	0	0	0	2 (20)	0	0	0	0
Day 84 - Desquamation	3 (33.3)	0	0	0	0	2 (20)	0	0	0	0
Day 84 - Edema	3 (33.3)	0	0	0	0	2 (20)	0	0	0	0
Day 84 - Erythema	3 (33.3)	0	0	0	0	2 (20)	0	0	0	0
Day 84 – Overall	3 (33.3)	0	0	0	0	2 (20)	0	0	0	0
Post-dose 1 - Desquamation	9 (100)	0	0	0	0	9 (90)	0	0	0	0
Post-dose 1 - Edema	9 (100)	0	0	0	0	9 (90)	0	0	0	0
Post-dose 1 - Erythema	9 (100)	0	0	0	0	9 (90)	0	0	0	0
Post-dose 1 – Overall	9 (100)	0	0	0	0	9 (90)	0	0	0	0
Post-dose 2 - Desquamation	9 (100)	0	0	0	0	9 (90)	0	0	0	0
Post-dose 2 - Edema	9 (100)	0	0	0	0	9 (90)	0	0	0	0
Post-dose 2 - Erythema	9 (100)	0	0	0	0	9 (90)	0	0	0	0
Post-dose 2 – Overall	9 (100)	0	0	0	0	9 (90)	0	0	0	0

Table S7. Objective Response Rate of MF patients in part B over time on treatment. SD = Stable disease 25% increase to <50% clearance in target lesions from baseline by CAILS), PD = Progressive disease (≥25% increase in target lesions from baseline by CAILS).

Treatment	Week	SD	PD
**Placebo**	Baseline	10 (100%)	0 (0%)
	Week 2	9 (90%)	1 (10%)
	Week 4	9 (100%)	0 (0%)
	Week 6	9 (100%)	0 (0%)
**Bimiralisib**	Baseline	9 (100%)	0 (0%)
	Week 2	9 (100%)	0 (0%)
	Week 4	9 (100%)	0 (0%)
	Week 6	9 (100%)	0 (0%)

Table S8. Pharmacodynamic assessments of target lesion(s) at day 42 compared to baseline.

	Bimiralisib (n=9) Baseline		Bimiralisib (n=9) Day 42		Placebo (n=10) Baseline		Placebo (n=9) Day 42	
	Mean	SD	Mean	SD	Mean	SD	Mean	SD
Erythema by 3D multispectral imaging (CIELAB *A)	19.59	3.90	19.88	4.31	21.83	5.13	21.13	6.59
Roughness by 3D multispectral imaging	25.81	12.60	18.77	7.30	22.03	8.80	16.84	10.13
Skin perfusion by LSCI (AU)	87.75	40.47	74.30	28.96	86.90	31.0	87.45	50.20
Blood flow at 0.3 mm by OCT (AU)	0.06	0.03	0.05	0.01	0.06	0.02	0.05	0.02
